# Thymosin Beta-4 Suppresses Osteoclastic Differentiation and Inflammatory Responses in Human Periodontal Ligament Cells

**DOI:** 10.1371/journal.pone.0146708

**Published:** 2016-01-20

**Authors:** Sang-Im Lee, Jin-Kyu Yi, Won-Jung Bae, Soojung Lee, Hee-Jae Cha, Eun-Cheol Kim

**Affiliations:** 1 Department of Dental Hygiene, School of Health Sciences, Dankook University, Cheonan, Republic of Korea; 2 Department of Conservative Dentistry, School of Dentistry, Kyung Hee University, Seoul, Republic of Korea; 3 Department of Oral and Maxillofacial Pathology and Research Center for Tooth and Periodontal Regeneration (MRC), School of Dentistry, Kyung Hee University, Seoul, Republic of Korea; 4 Department of Oral Physiology, School of Dentistry, Kyung Hee University, Seoul, Republic of Korea; 5 Department of Parasitology and Genetics, College of Medicine, Kosin University Busan, Republic of Korea; Charles P. Darby Children's Research Institute, UNITED STATES

## Abstract

**Background:**

Recent reports suggest that thymosin beta-4 (Tβ4) is a key regulator for wound healing and anti-inflammation. However, the role of Tβ4 in osteoclast differentiation remains unclear.

**Purpose:**

The purpose of this study was to evaluate Tβ4 expression in H_2_O_2_-stimulated human periodontal ligament cells (PDLCs), the effects of Tβ4 activation on inflammatory response in PDLCs and osteoclastic differentiation in mouse bone marrow-derived macrophages (BMMs), and identify the underlying mechanism.

**Methods:**

Reverse transcription-polymerase chain reactions and Western blot analyses were used to measure mRNA and protein levels, respectively. Osteoclastic differentiation was assessed in mouse bone marrow-derived macrophages (BMMs) using conditioned medium (CM) from H_2_O_2_-treated PDLCs.

**Results:**

Tβ4 was down-regulated in H_2_O_2_-exposed PDLCs in dose- and time-dependent manners. Tβ4 activation with a Tβ4 peptide attenuated the H_2_O_2_-induced production of NO and PGE_2_ and up-regulated iNOS, COX-2, and osteoclastogenic cytokines (TNF-α, IL-1β, IL-6, IL-8, and IL-17) as well as reversed the effect on RANKL and OPG in PDLCs. Tβ4 peptide inhibited the effects of H_2_O_2_ on the activation of ERK and JNK MAPK, and NF-κB in PDLCs. Furthermore, Tβ4 peptide inhibited osteoclast differentiation, osteoclast-specific gene expression, and p38, ERK, and JNK phosphorylation and NF-κB activation in RANKL-stimulated BMMs. In addition, H_2_O_2_ up-regulated Wnt5a and its cell surface receptors, Frizzled and Ror2 in PDLCs. Wnt5a inhibition by Wnt5a siRNA enhanced the effects of Tβ4 on H_2_O_2_-mediated induction of pro-inflammatory cytokines and osteoclastogenic cytokines as well as helping osteoclastic differentiation whereas Wnt5a activation by Wnt5a peptide reversed it.

**Conclusion:**

In conclusion, this study demonstrated, for the first time, that Tβ4 was down-regulated in ROS-stimulated PDLCs as well as Tβ4 activation exhibited anti-inflammatory effects and anti-osteoclastogenesis *in vitro*. Thus, Tβ4 activation might be a therapeutic target for inflammatory osteolytic disease, such as periodontitis.

## Introduction

Bone loss associated with inflammatory diseases, such as rheumatoid arthritis, periodontal disease, and osteoporosis, and elevated osteoclast activity leads to bone destruction [1]. The most common osteolytic disease, periodontitis, is a multi-factorial irreversible and cumulative condition, initiated and propagated by bacteria and host factors [2]. Destruction of peridontal tissue is mediated *via* the expression of various tissue-destructive enzymes or inflammatory mediators such as interleukins-1 (IL-1), IL-6 and IL-8, tumor necrosis factor- α (TNF- α), nitric oxide (NO), and prostaglandin E_2_ (PGE_2_) [2]. Receptor activator of nuclear factor-kappa B (NF-κB) ligand (RANKL) and osteoprotegerin (OPG) are critical for homeostatic control of osteoclast activity, suggesting that they have vital roles in the progression of bone loss in periodontitis [3, 4]. Therefore, resolution of inflammation and blocking osteoclast differentiation might be a potential therapeutic approach for the prevention and treatment of osteolytic inflammatory disease, such as periodontitis [5].

Thymosin beta-4 (Tβ4) is a water-soluble, 43-amino acid, and 4.9 kDa protein that was originally isolated from bovine thymus [6]. Since Tβ4 is the major actin-sequestering molecule in eukaryotic cells and is found in all cells [7], Tβ4 has multiple diverse cellular functions, including tissue development, migration, angiogenesis, and wound healing [7]. We previously reported that Tβ4-overexpressing transgenic mice, using a construct on the skin-specific keratin-5 promoter, have abnormal tooth development and enhanced stimulation of hair growth [8]. Moreover, exogenous Tβ4 has anti-inflammatory effects in the bleomycin-induced mouse model of lung fibrosis [9], tooth extraction sockets in rats [10], rat model of myocardial ischemia [11], corneal wound healing [12], wound healing of rat palatal mucosa [13], *in vitro* model of cultured human gingival fibroblasts [14], and cardiac fibroblasts [15]. However, the effects of Tβ4 over expression or inhibition on differentiation are controversial. Exogenous β4 peptide inhibited osteogenic differentiation but facilitated adipogenic differentiation in human bone marrow-derived-mesenchymal stem cells (MSCs) [16]. In contrast, Tβ4 inhibition by Tβ4 siRNA attenuated odontoblastic differentiation in the odontoblast-like cells, MDPC-23 [17]. Moreover, we recently demonstrated that odontoblastic differentiation was enhanced by activation of Tβ4 by Tβ4 peptide but was decreased by Tβ4 siRNA in human dental pulp cells (HDPCs) [18]. However, the effects of Tβ4 on osteoclastic differentiation have not been reported.

Moreover, Tβ4 concentration revealed wide variability, and it decreased in the gingival crevicular fluid (GCF) as periodontal disease progressed [19]. In contrast, Tβ4 mRNA expression was 3.76 fold higher in periodontitis-affected gingival tissue, compared with healthy individuals’ tissue obtained from public microarray data (GEO assession: GSE 23586) [20]. However, the Tβ4 mRNA level did not change in the periodontal-diseased gingival tissue (arbitrary units; 6.249) when compared with healthy tissue (arbitrary units; 6.242) (GEO assession: GSE 10334) [21]. Although Tβ4 exerts anti-inflammatory effects *in vivo* and *in vitro*, the precise role of Tβ4 in the inflammatory response remains unclear.

Therefore, this study was designed to investigate whether Tβ4 was up-regulated in patients with periodontitis, and this study was also designed to investigate whether Tβ4 inhibition or activation suppressed the osteoclastic differentiation in mouse bone marrow-derived macrophages (BMMs) and inflammatory response in periodontal ligament cells (PDLCs) as well as on their signaling pathways.

## Materials and Methods

### Cell culture

Established immortalized human PDLCs [22] that maintain the characteristics of primary PDLCs by transfecting human telomerase reverse transcriptase (*hTERT*) were used. These cell line were kindly provided by Professor Takashi Takata (Hiroshima University, Japan). Cells were cultured in α-MEM supplemented with 10% FBS, 100 U/mL penicillin, and 100 μg/mL streptomycin in a humidified atmosphere of 5% CO_2_ at 37°C. For the experiments, the cells were seeded into culture dishes and then cultured in α-MEM containing 10% FBS for 2 days until 70% confluent, and, then, the media was replaced by serum-free medium in order to minimize any serum-induced effects on PDLCs. Subsequently, the cells were exposed to H_2_O_2_ and human Tβ4 peptide (RegeneRx Biopharmaceuticals Inc., Rockville, MD). All treatments were performed in triplicate and approved by the local ethics committee.

### Quantification of nitric oxide (NO) and prostaglandin E_2_ levels

Total NO production was determined as the nitrite concentration in the culture medium using a spectrophotometric assay based on the Griess reaction. The concentrations of PGE_2_ in culture supernatants were determined using an ELISA kit (R&D Systems, Minneapolis, MN, USA), according to the manufacturer’s recommendations.

### Cell viability assay

The cytotoxicity was determined by the 3-(4,5-dimethylthiazolyl-2-yl)-2,5-diphenyltetrazolium bromide (MTT) assay. Cells seeded on 96-well microplates at 1×10^4^ cells/well were incubated with H_2_O_2_ and Tβ4, for 48 hr. Medium was removed and then incubated with 100 μl MTT assay solution for 4 h. Absorbance was measured in an ELISA reader at 595 nm.

### Osteoclast differentiation

Mouse bone marrow macrophage (BMMs) of 5-week-old female ICR mice (Charles River Laboratories, Seoul, South Korea) were used as previously described [23]. Animals were maintained in accordance with the National Institute of Toxicological Research of the Korea Food and Drug Administration guideline for the humane care and use of laboratory animals Institutional Animal Care and Use Committee (IACUC) approval was obtained from Kyung Hee University (Seoul, Korea). Briefly, bone marrow of tibiae and femurs of mice were flushed with α-MEM. After removing erythrocytes with hypotonic buffer, cells were cultured in α-MEM containing 10% FBS for 24 h and adherent cells were discarded. Non-adherent bone marrow cells were transferred onto 100-mm non-coated petri dishes at 5×10^6^ cells per dish and cultured in the presence of M-CSF (30 ng/ml) for 3 days. Condition medium (CM) was obtained from HPDLCs treated with 200 μM H_2_O_2_ or Tβ4 (0.5, 1 and 5 μg/mL) for 2 days. To evaluate the osteoclastogenic activity of CM from HPDLCs, we added the CM mixture (60% CM plus 40% fresh α-MEM without M-CSF or RANKL) or rh-Tβ4 to pre-osteoclast-stage cells and further cultured the cells for up to 5 days to achieve mature osteoclast differentiation BMMs (1.5 × 10^5^ cells/well) and PDLCs (1.5 × 10^4^ cells/well) were co-cultured for 7 days in the presence of M-CSF (30 ng/ml), RANKL (100 ng/mL), H_2_O_2_ (200 μM) or Tβ4 (0.5, 1 and 5 μg/mL) in α-MEM, supplemented with10% in 48-well plates under 5% CO_2_ atmosphere.

### TRAP staining and activity assay

Osteoclast differentiation was assessed by tartrate-resistant acid phosphatase (TRAP) staining and activity. After 5 days of culture, cells were stained for TRAP kit using a leukocyte acid phosphatase kit (Sigma Aldrich, St Louis, MO, USA). Cells with three or more nuclei were counted as multinucleated mature osteoclasts. To measure TRAP activity, cells were fixed with 10% formalin for 10 min and 95% ethanol for 1 min, and then 100 μl of citrate buffer (50 mM, pH 4.6) containing 10 mM sodium tartrate and 5 mM p-nitrophenylphosphate (Sigma-Aldrich) was added to the wells containing fixed cells in the 48-well plates. After incubation for 1 h, enzyme reaction mixtures in the wells were transferred to new plates containing an equal volume of 0.1 N NaOH. Absorbance was measured at 410 nm using a microplate reader.

### Tβ4 or Wnt5a siRNA transfection

Silencing of the Tβ4 or Wnt5a gene was achieved by transfecting cells with small interfering RNA (siRNA). Cells were transfected with Tβ4 or Wnt5a siRNAs (30 nM) for 24 hours using Lipofectamine 2000 (Invitrogen, Carlsbad, CA, USA) according to the manufacturer's instructions. Cells were transfected with Silencer negative control siRNA using the same protocol.

### RNA extraction, reverse transcriptase PCR

Total RNA was extracted from cells using Trizol (Invitrogen, Carlsbad, CA, USA) according to the manufacturer’s instructions. Reverse-transcription (RT)-PCR was performed using oligo deoxythymidine primer (Roche Diagnostics, Mannheim, Germany) in 20 μl volumes at 42°C for 60 min. The RT-PCR reaction was done with 1 μg of total RNA, 1 μl of 20 μM oligo dT primer, and 18 μl of reaction mixture by *AccuPower* RT/PCR PreMix (Bioneer, Daejeon, Korea). Then, PCR was performed in a 20 μl total mixture volume for 25 cycles at 95°C for 1 min, 55°C for 1 min, and 72°C for 1 min. Primer sequences are detailed in Table 1. PCR products were subjected to electrophoresis on 1.5% agarose gels and visualized with ethidium bromide.

**Table 1:** Reverse transcriptase-polymerase chain reaction (RT-PCR) primers and conditions.

Genes	Forward primer (5'-3')	Reverse primer (5'-3')	Annealing temperature (°C)	Cycle number	Product size (bp)
Tβ4	5'-CGCAGACCAGACTTCGCTCGTAC-3'	5'-TCCTTCCCTGCCAGCCAGATAGAT-3'	58	30	389
COX-2	5'-GGAGAGACTATCAAGATAGTGATC-3'	5'-ATGGTCAGTAGACTTTTACAGCTC-3'	60	38	860
iNOS	5'-CCCTTCCGAAGTTTCTGGCAGCAGC-3'	5'-GGCTGTCAGAGAGCCTCGTGGCTTTGG-3'	61	35	497
TNF-α	5'- GGAAGACCCCTCCCA GAT AG -3'	5'- CCCCAGGGACCTCTCTCTAA -3'	52	35	413
IL-1β	5'- GGATATGGAGCAACAAGTGG -3'	5'- ATGTACCAGTTGGGGAACTG -3'	60	35	288
IL-6	5'-ATGAACTCCTTCTCCACAAGC-3'	5'-CTACATTTGCCGAAGAGCCC-3'	55	34	639
IL-8	5'-ATGACTTCCAAGCTGGCCGTGGCT-3'	5'-TCTCAGCCCTCTTCAAAAACTTCTC-3'	62	25	289
IL-17	5'-CGATGACTCCTGGGAAGACCTC-3'	5'-GTGTGGGCTCCCCAGAGCTCTTA-3'	62	30	524
RANKL	5'-CCAGCATCAAAATCCCAAGT-3'	5'-CCCCTTCAGATGATCCTTC-3'	56	35	605
OPG	5'-TCAAGCAGGAGTGCAATCG-3'	5'-AGAATGCCTCCTCACACAGG-3'	59	31	342
Wnt5a	5'-CCGCGAGCGGGAGCGCATCCA CGCC-3'	5'-GCCACATCAGCCAGGTTGTACACC-3'	54	32	114
Ror2	5'-ATCCAAGACCTGGACACAACAGA-3'	5'-GAACCCCAGTGGCAGTGATG-3'	60	30	85
Frizzled	5'-GCGAAGCCCTCATGAACAA-3'	5'-TCCGTCCTCGGAGTGGTTCT-3'	60	30	116
GAPDH	5'-CGGAGTCAACGGATTTGGTCGTAT-3'	5'-AGCCTTCTCCATGGTGGTGAAGAC-3'	62	25	306
Mouse Cathepsin K	5'-TGGATCTTTTAGCGTCTTGTTCC-3'	5'-CCACGCTTGAGACAGGCTTA-3'	60	30	226
Mouse Calcitonin receptor	5'-TGGTTGAGGTTGTGCCCAATGGAGA-3'	5'-CTCGTGGGTTTGCCTCATCTTGGTC-3'	65	32	503
Mouse NFATc1	5'-TCGAGTTCGATCAGAGCGG-3'	5'-TGGCTGAAGGAACAGCTGAG-3'	59	34	168
Mouse RANK	5'-TCCTACCTCCGACAGTGTGT-3'	5'-CCGTATCCTTGTTGAGCTGC-3'	58	31	266
Mouse c-fms	5'-GGAAGAGGAGCCAGTGCAAC-3'	5'-AAGAGTGGGCCGGATCTTTG-3'	60	30	450
Mouse GAPDH	5'-GAGAGTGTTTCCTCGTCCCG-3'	5'-ACTGTGCCGTTGAATTTGCC-3'	59	28	201

### Western blotting

Treated cells were washed with PBS and cytosolic protein extracts were prepared using 1X cell lysis buffer (Santa Cruz Biotechnology, CA) supplemented with protease inhibitor cocktail. Protein concentrations were determined using the Bradford assay (Bio-Rad, CA, USA) as per the manufacturer's protocol. Aliquots of protein lysates were separated on sodium dodecyl sulfate–10% polyacrylamide gels and Western blotting was performed. The proteins were transferred onto a polyvinylidene difluoride membrane (Bio-Rad, CA, USA) in transfer buffer (20 mm Tris, 150 mm glycine, 20% methanol, pH 8.0; TBS-T) at 4°C and 100 V for 1 hour. The membrane was blocked with 5% dry milk in TBS-T for 1 hour at room temperature and incubated with primary antibodies (1:1000) and horseradish peroxidase (HRP)-conjugated secondary antibodies. Protein bands were detected using an enhanced chemiluminescence (ECL) system (Amersham Biosciences, Backinghamshire, UK).

### Statistical analysis

Differences among groups were analyzed using a one-way analysis of variance combined with the Bonferroni test. The relative intensities of mRNA and protein bands were assayed using Quantity-One software (Bio-Rad Co., Hercules, CA, USA); results were normalized to the mRNA and protein levels of beta-actin. All values were expressed as mean ± standard deviation. Differences were considered significant at *p* < 0.05.

## Results

### Effects of ROS on Tβ4 mRNA and protein expression in human PDLCs

**Fig 1:**
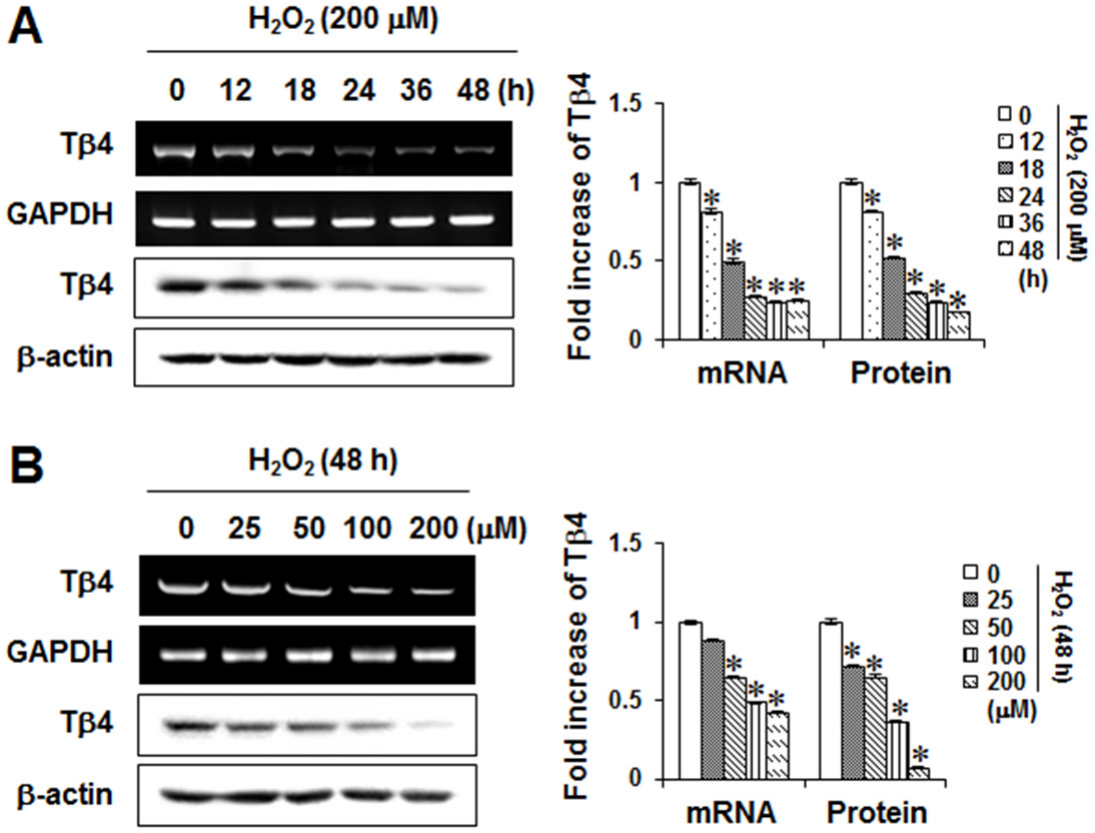
Effects of H_2_O_2_ on mRNA and protein expression of Tβ4 in PDLCs. Cells were incubated for 48 hours with the indicated times with 200 μM H_2_O_2_ (A) and the indicated concentrations of H_2_O_2_ (B) for 48 hours. The mRNA and protein expressions were examined by RT-PCR and Western blotting, respectively. Data were representative of three independent experiments. The bar graph shows the fold increase in protein or mRNA expression compared with control cells. * Statistically significant differences compared with the control, *p*<0.05.

As reactive oxygen species (ROS) have been implicated in the pathogenesis of periodontitis [24], we examined whether H_2_O_2_ could down-regulate or up-regulate Tβ4 expression in PDLCs. As shown in Fig 1A and 1B, Tβ4 mRNA and protein expressions were down-regulated by H_2_O_2_ in a time- and concentration-dependent manner. Because maximal Tβ4 mRNA and protein expressions were achieved with 200 μM H_2_O_2_ within 48 hours in PDLCs, this concentration was used in subsequent experiments.

### Effects of Tβ4 peptide on H_2_O_2_-induced inflammatory response in PDLCs

**Fig 2:**
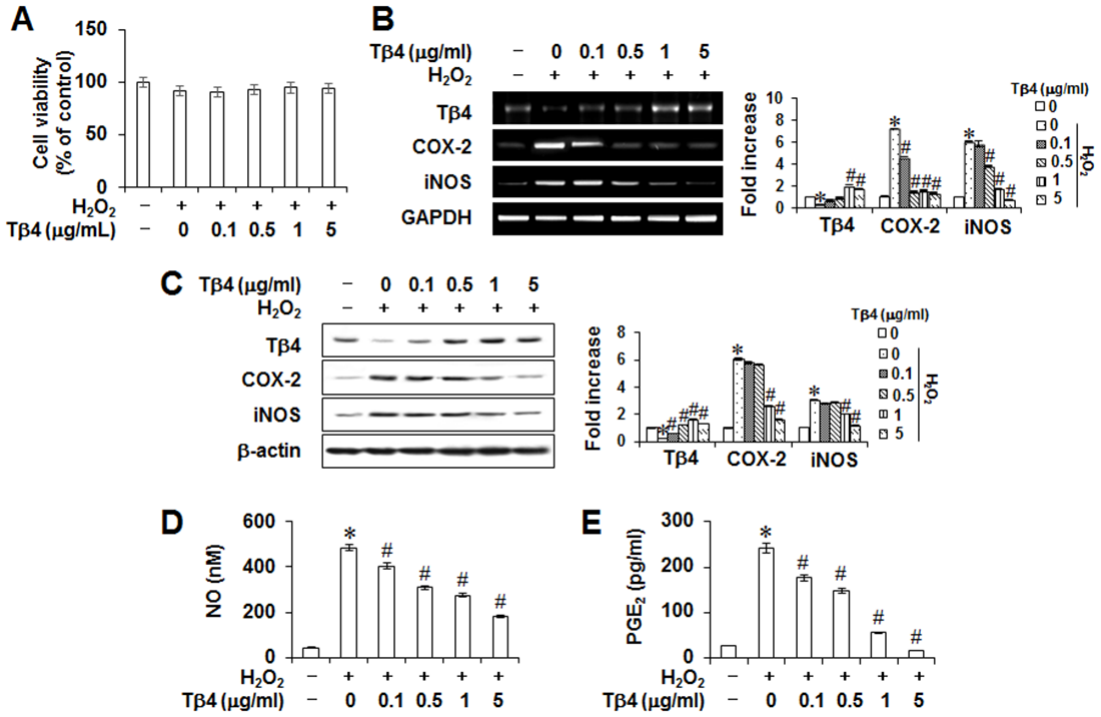
Effect of Tβ4 peptide on H_2_O_2_-induced cytotoxicity (A), Tβ4, inducible nitric oxide (NO) synthase (iNOS) and cyclooxygenase-2 (COX-2) mRNA and protein expressions (B, C), NO and prostaglandin E_2_ (PGE_2_) secretion (D, E) in PDLCs. Cells were pretreated with indicated concentrations of Tβ4 peptide for 2 hours and then incubated with 200 μM H_2_O_2_ for 48 hours (A-E). Cell viability was measured by MTT assay (A). Protein and mRNA expressions were assessed by RT-PCR (B) and Western blot analysis (C), respectively. The production of NO (D) and PGE_2_ (E) were measured by Griess reaction and ELISA, respectively. Data replicated the quantifications of cytotoxicity, NO, and PGE_2_ with the standard deviation of at least three experiments (n = 4). The bar graph shows the fold increase in protein or mRNA expression compared with control cells. * Statistically significant differences compared with the control, *p*<0.05. # Statistically significant difference compared with the H_2_O_2_—treated group.

To determine the effects of Tβ4 peptide and H_2_O_2_ on cytotoxicity, its cell viability was evaluated. A 48-h exposure to 0.1–5 μg/mL Tβ4 peptide did not affect H_2_O_2_-mediated cell viabilities (Fig 2A). In order to examine whether Tβ4 peptide suppressed ROS-induced inflammatory mediators, the ability of Tβ4 peptide on production of NO and PGE_2_, and expressions of COX-2 and iNOS were measured by RT-PCR, Western blot, and ELISA. Pretreatment with Tβ4 peptide dose-dependently inhibited H_2_O_2_-induced mRNA and protein expressions of COX-2 and iNOS, and NO and PGE_2_ production (Fig 2B–2E).

**Fig 3:**
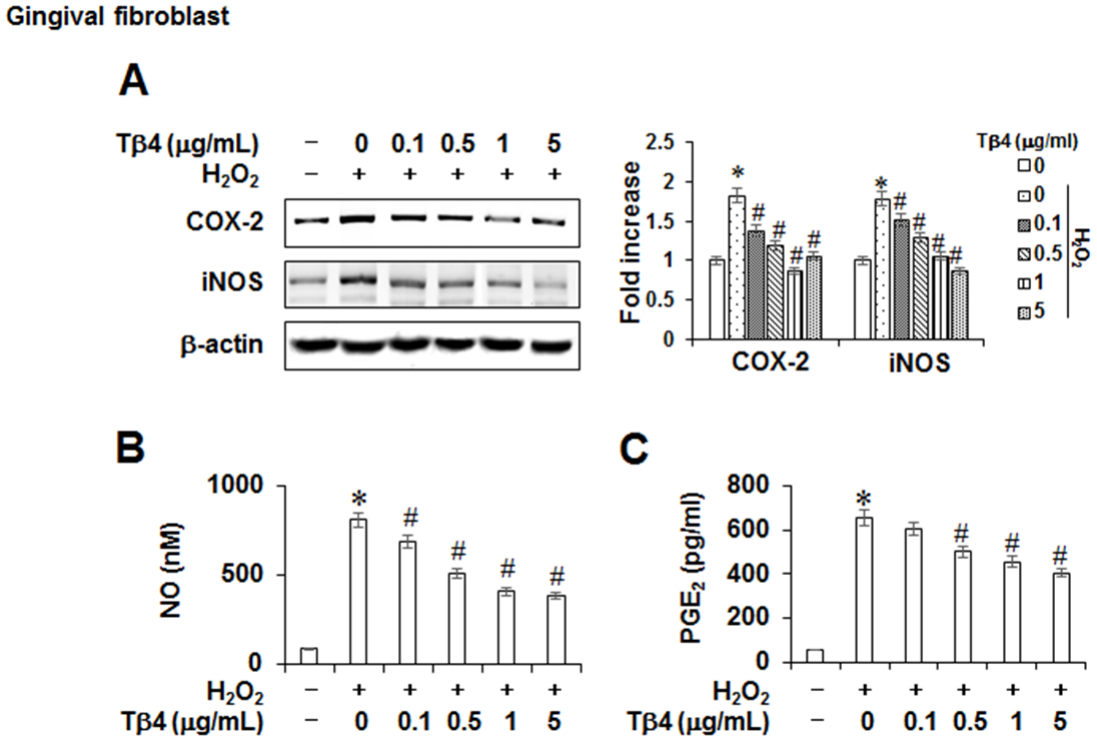
Anti-inflammatory effect of Tβ4 peptide in gingival fibroblasts. Cells were pretreated with indicated concentrations of Tβ4 peptide for 2 hours and then incubated with 200 μM H_2_O_2_ for 48 hours (A-C). Protein expressions were assessed by Western blot analysis (A). The production of NO (B) and PGE_2_ (C) were measured by Griess reaction and ELISA, respectively. Data replicated the quantifications of NO and PGE_2_ with the standard deviation of at least three experiments (n = 4). The bar graph shows the fold increase in protein expression compared with control cells. * Statistically significant differences compared with the control, *p*<0.05. # Statistically significant difference compared with the H_2_O_2_—treated group.

To examine whether suppression of inflammatory events by Tβ4 is specific to PDLCs, the anti-inflamamtory effects of Tβ4 peptide in gingival fibroblasts were verified. Tβ4 peptide also inhibited H_2_O_2_-induced mRNA and protein expressions of COX-2 and iNOS, and NO and PGE_2_ production in gingival fibroblasts (Fig 3A–3C).

**Fig 4:**
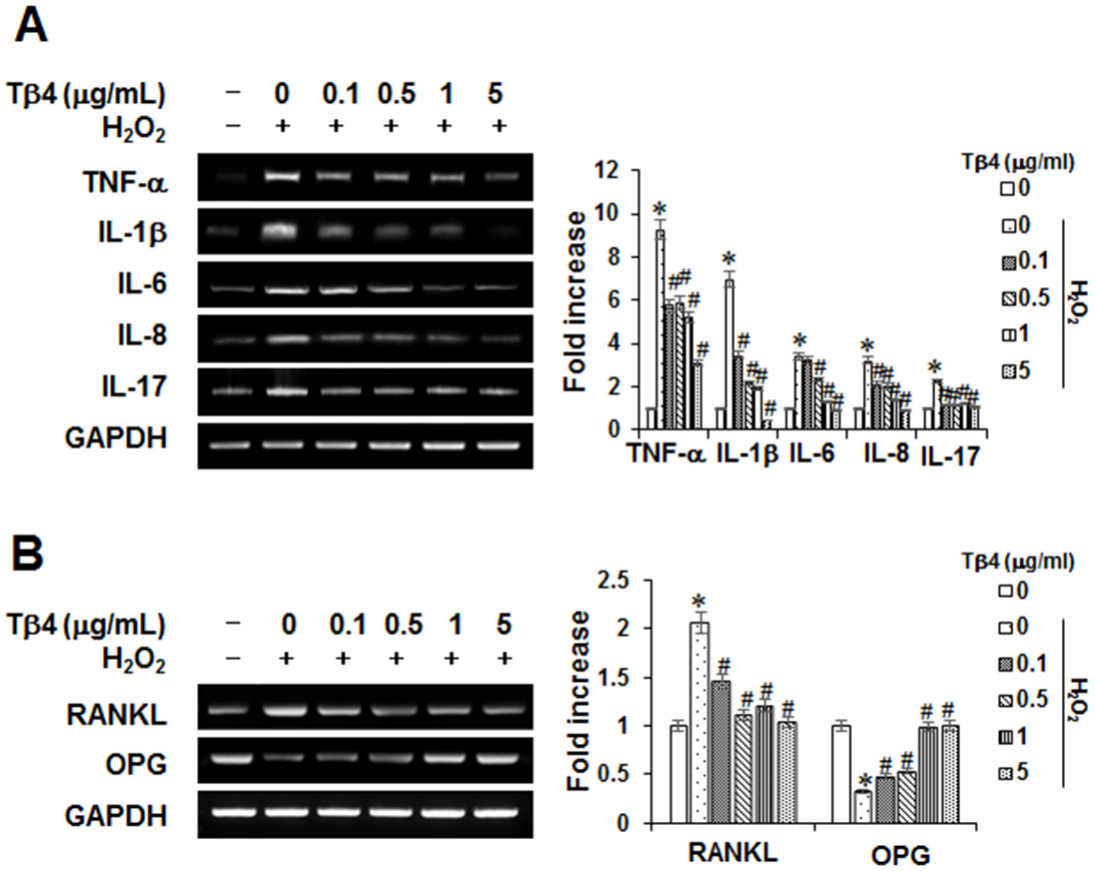
Effect of Tβ4 peptide on H_2_O_2_-induced osteoclastogenic cytokines (A) and osteoclastogenic factors (B) in PDLCs. Cells were pretreated with indicated concentrations of Tβ4 peptide for 2 hours and then incubated with 200 μM H_2_O_2_ for 48 hours (A, B). The mRNAs expression was examined by RT-PCR analysis. This data were representative of three independent experiments. The bar graph shows the fold increase in mRNA expression compared with control cells. * Statistically significant differences compared with the control, *p*<0.05.

To further determine the potential anti-inflammatory effects of Tβ4 activation, expressions of proinflammatory or osteoclastogenic cytokines were measured by RT-PCR (Fig 4A). The TNF-α, IL-1β, IL-6, IL-8, and IL-17 mRNA levels increased in the H_2_O_2_- stimulated PDLCs, and these increases were significantly decreased in a concentration-dependent manner by treatment with the Tβ4 peptide. Since receptor activator of NF-κB ligand (RANKL) and osteoprotegerin (OPG) are two important osteoclastogenic factors, we next explored the effects of Tβ4 peptide on RANKL and OPG expressions in PDLCs. Tβ4 peptide reduced H_2_O_2_-stimulated up-regulation of RANKL, with a reciprocal increase in OPG mRNA in a dose-dependent manner (Fig 4B).

### Effects of Tβ4 peptide on H_2_O_2_-induced signal transduction pathway in PDLCs

**Fig 5:**
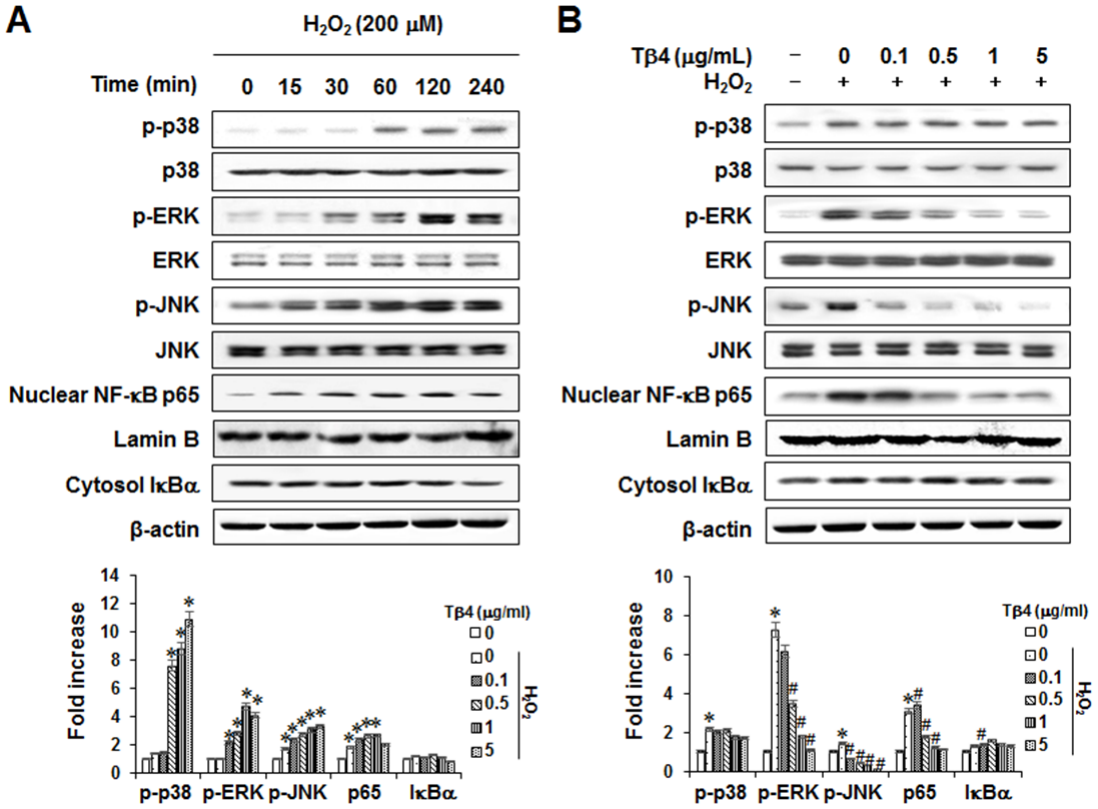
Effect of H_2_O_2_-induced MAPK and NF-κB signaling pathways (A) and effect of Tβ4 peptide on H_2_O_2_-induced MAPK and NF-κB activation (B) in PDLCs. Cells were incubated with 200 μM H_2_O_2_ for indicated times (A). Cells were pretreated with indicated concentrations of Tβ4 peptide (0.1–5 μg/mL) for 2 hours and then incubated with 200 μM H_2_O_2_ for 60 minutes (B). Data were representative of three independent experiments. The bar graph shows the fold increase in protein expression compared with control cells * Statistically significant differences compared with the control, *p*<0.05. # Statistically significant difference compared with the H_2_O_2_—treated group.

To investigate the effect of Tβ4 peptide on H_2_O_2_-induced signaling cascades, the activation states of three mitogen-activated protein kinases (MAPKs; p38, c-Jun N-terminal kinase [JNK] and extracellular signal-related kinase [ERK]) as well as NF-κB p65 were examined in PDLCs. H_2_O_2_ treatment induced the phosphorylation of p38, ERK, and JNK MAPK(s) and the nuclear translocation of NF-κB p65 (Fig 5A). Treatment of cells with Tβ4 peptide blocked H_2_O_2_-induced nuclear translocation of NF-κB p65 and phosphorylation of ERK and JNK (Fig 5B).

### Effects of Tβ4 peptide on osteoclastogenesis

**Fig 6:**
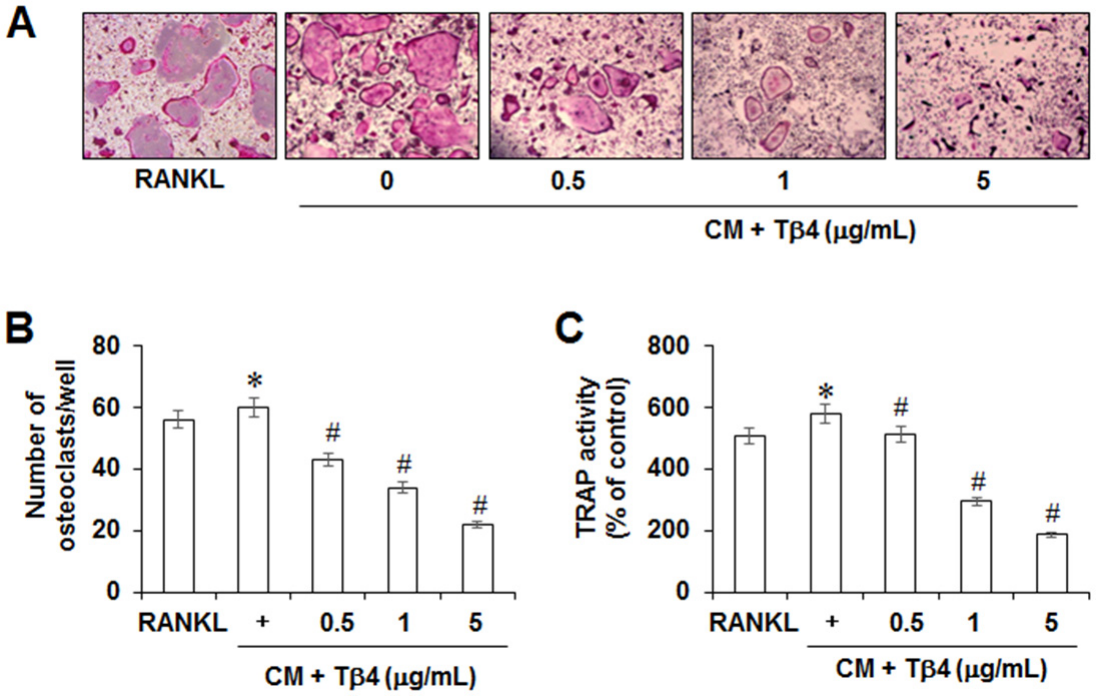
Indirect effects of Tβ4 peptide on RANKL-induced osteoclastogenesis in mouse BMMs (A-C). Cells were pretreated with indicated concentrations of Tβ4 peptide for 2 hours, post-incubated with 200 μM H_2_O_2_ for 48 hours, and then conditioned medium (CM) was collected. Mouse BMMs were cultured with CM in the presence of M-CSF (30 ng/mL) and RANKL (100 ng/mL), as described in Materials and methods. After 5 days, cells were fixed and stained for TRAP as a marker of osteoclasts (A), and the number of TRAP-positive multinucleated cells (MNCs) was scored (B). TRAP osteoclast activity was assayed using the TRAP cytochemical stain technique (C). * Statistically significant differences compared with the control, *p*<0.05. The data presented were representative of three independent experiments.

To evaluate the indirect effect of Tβ4 peptide on RANKL-induced osteoclastogenesis, mouse BMMs were incubated with RANKL and CM, prepared from HPDLCs treated with H_2_O_2_ and different concentrations of Tβ4, and allowed to differentiate into osteoclasts. As shown in Fig 6, Tβ4 peptide dose-dependently decreased the number of osteoclasts and TRAP activity. To determine whether the reduction in osteoclast generation by Tβ4 could be due to effects of Tβ4 peptide on viability of the BMMs, a cytotoxicity assay was performed. The viability of BMMs was not significantly affected by Tβ4 peptide (data not shown).

**Fig 7:**
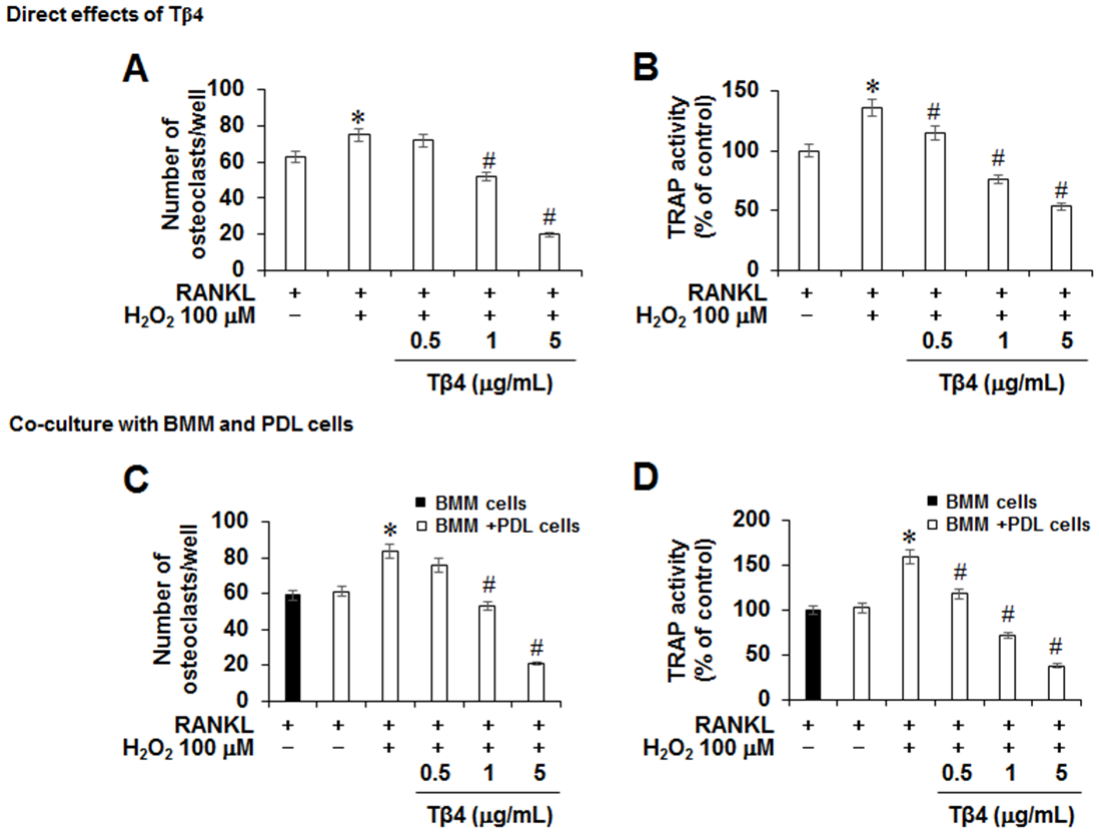
Direct (A, B) or co-cultured (C, D) effects of Tβ4 peptide on RANKL-induced osteoclastogenesis in mouse BMMs (A-C). A and B; Mouse BMMs were cultured with 200 μM H_2_O_2_ and indicated concentrations of Tβ4 peptide in the presence of M-CSF (30 ng/mL) and RANKL (100 ng/mL). C and D; PDLCs were co-cultured with mouse BMMs in the presence of M-CSF, RANKL, 200 μM H_2_O_2_, and indicated concentrations of Tβ4 peptide. To monitor osteoclast differentiation, both TRAP activity and the number of TRAP multinucleated cells were examined. * Statistically significant difference compared with control, *p*<0.05. The data presented were representative of three independent experiments.

To determine the direct effect of Tβ4 peptide on osteoclastogenesis, mouse BMMs were directly exposed to Tβ4 peptide. Direct treatment with Tβ4 peptide also reduced the number of multinucleated TRAP-positive cells and TRAP activity in a dose-dependent manner (Fig 7A and 7B). Since Tβ4 downregulated H_2_O_2_-induced various cytokines expression, the indirect effect of Tβ4 on osteoclast formation through PDLC cells using co-culture system were investigated. After addition of Tβ4 peptide to the BMMs-PDLCs co-culture, the number of osteoclast and TRAP activity were also significantly decreased (Fig 7C and 7D).

### Effects of Tβ4 peptide on signaling pathways of osteoclastogenesis

**Fig 8:**
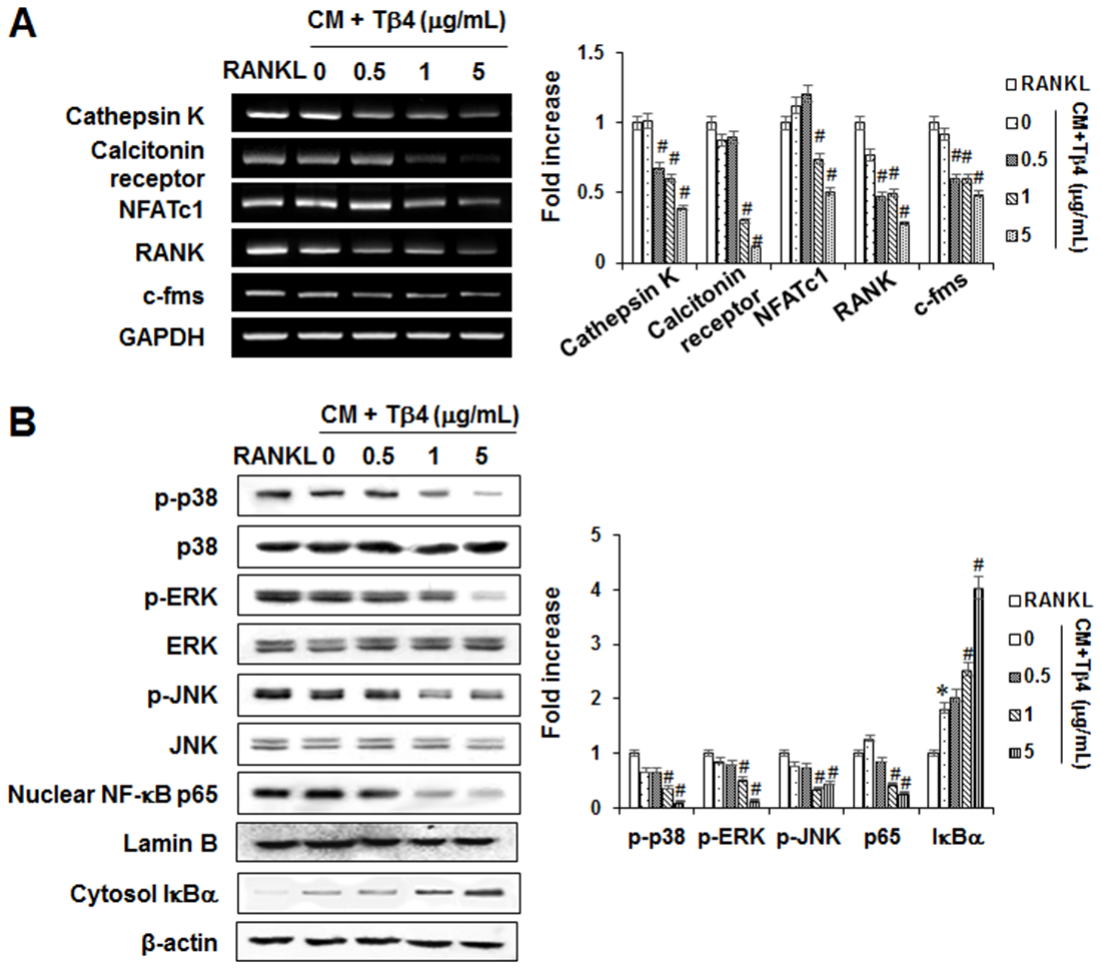
Effects of Tβ4 peptide on the functional changes of osteoclastogenesis (A) and MAPK and NF-κB signaling pathways in mouse BMMs. Mouse BMMs were cultured with M-CSF (30 ng/mL) and RANKL (100 ng/mL) or CM collected from PDLCs for 5 days (A) and 60 minutes (B). The mRNAs expression was determined by PCR analysis (A). The phosphorylation of MAPKs (p38, JNK, and ERK), and activation of NF-κB were determined by Western blot analysis (B). Data were representative of three independent experiments. The bar graph shows the fold increase in protein or mRNA expression compared with control cells * Statistically significant differences compared with the control, *p*<0.05.

To analyze the functional changes of osteoclastogenesis induced by Tβ4 peptide treatment, total RNA from BMMs was collected and RT-PCR for osteoclast-specific gene expression was performed. Tβ4 peptide reduced RANKL-induced cathepsin K, calcitonin receptor (CR), NFATc1, RANK, and c-fms up-regulations in a dose-dependent manner (Fig 8A).

To determine whether MAPK and NF-κB signaling pathways were involved in the anti-osteoclastogenic function of Tβ4, the effect of Tβ4 peptide on the phosphorylation levels of ERK, JNK, and p38 MAPK(s) as well as the nuclear translocation of NF-κB p65 in RANKL-stimulated BMMs were examined. As shown in Fig 8B, Tβ4 peptide inhibited the RANKL-induced phosphorylation of p38, ERK, and JNK and nuclear translocation of NF-κB p65.

### Role of the Wnt-5a pathway in anti-inflammatory response and anti-osteoclastogenesis by Tβ4

**Fig 9:**
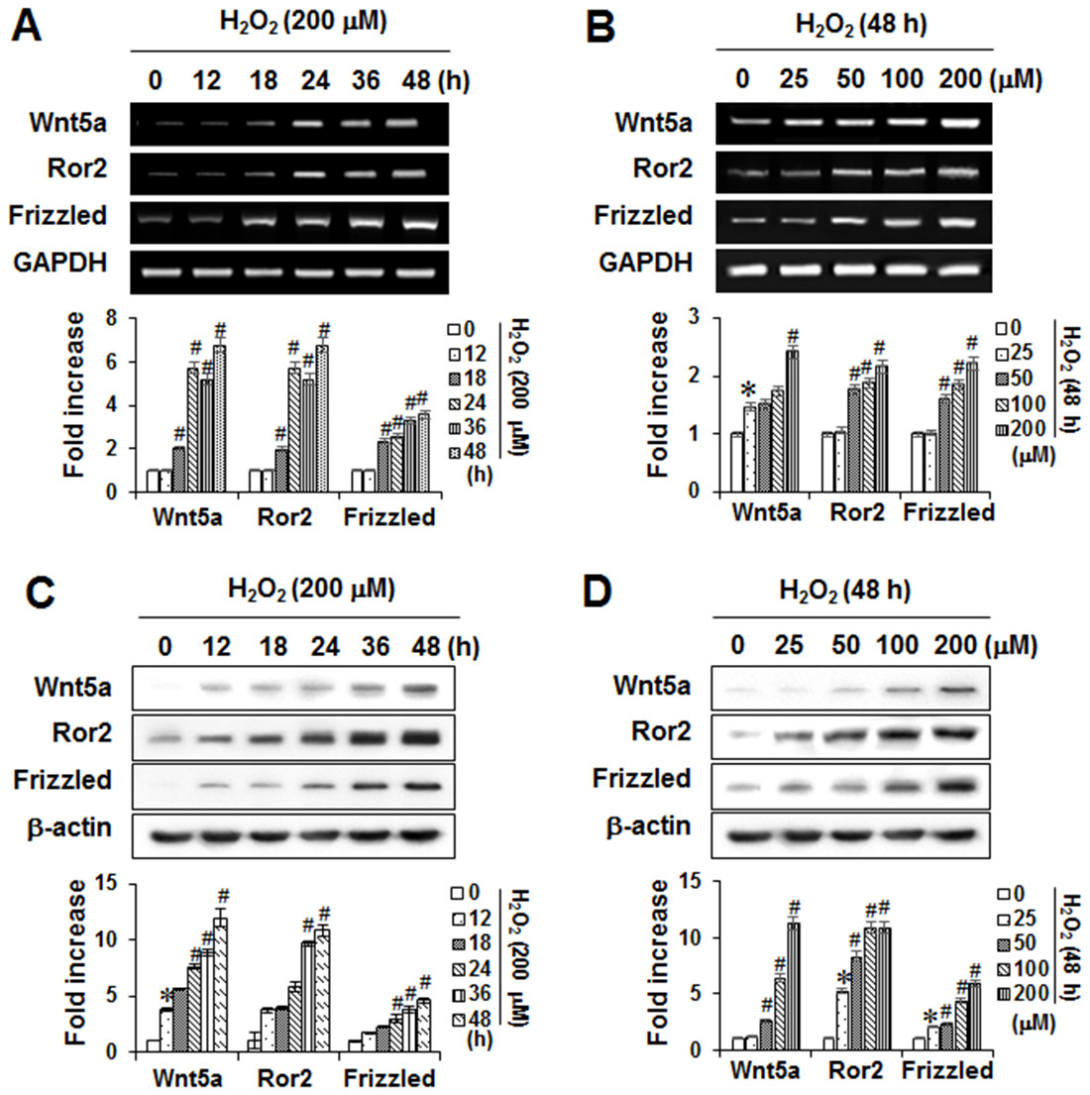
Effects of H_2_O_2_ on the expression of Wnt5a and its cell surface receptors in PDLCs. The mRNA and protein expressions were determined by PCR analysis (A) and Western blot analysis (B), respectively. The bar graph shows the fold increase in protein or mRNA expression compared with control cells * Statistically significant differences compared with the control, *p*<0.05. The data presented were representative of three independent experiments.

Since Wnt5a expression is associated with rheumatoid arthritis and periodontitis [25, 26], expression of Wnt5a and its cell surface receptors, Frizzled and receptor tyrosine kinase-like orphan receptor 2 (Ror2), were examined. As shown in Fig 9A–9D, mRNA and protein expressions of Wnt5a and its receptors were increased by H_2_O_2_ in a time- and dose-dependent manner.

**Fig 10:**
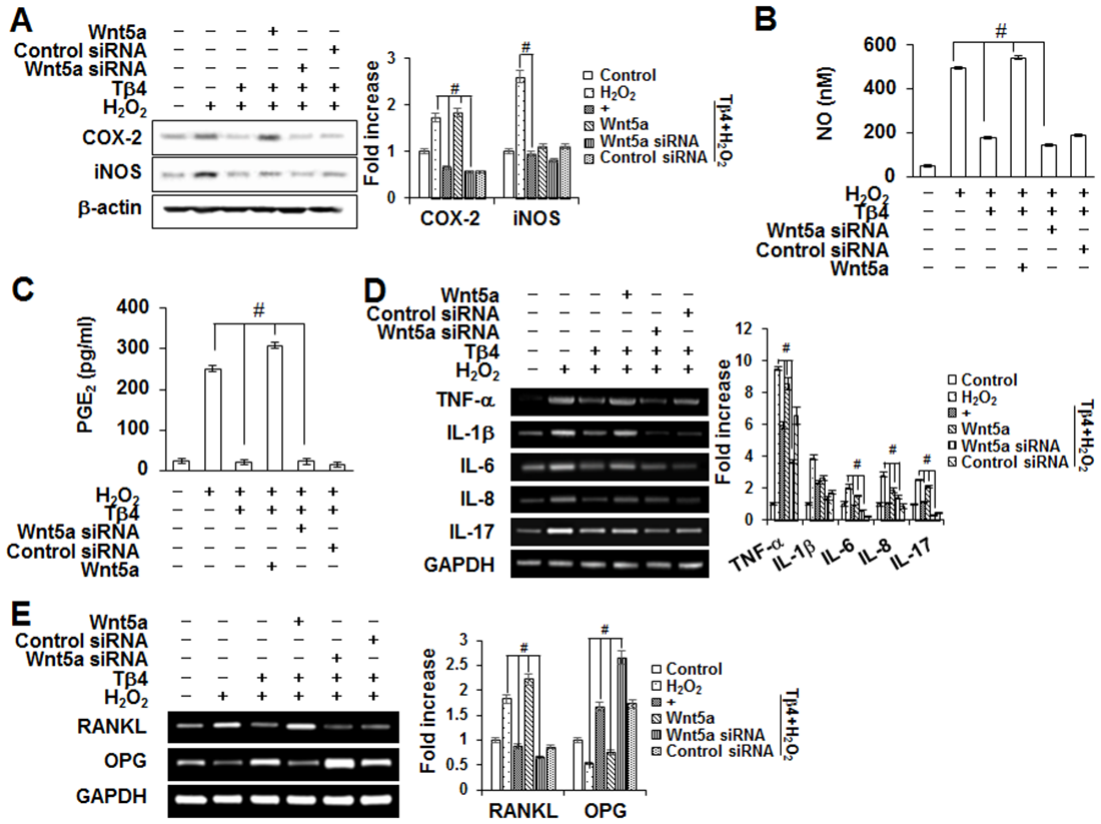
Effects Wnt5a siRNA and Wnt5a peptide on the Tβ4 peptide-mediated inhibition of iNOS and COX-2 expressions (A), NO and PGE_2_ secretions (B, C), pro-inflammatory cytokines production (D) and osteoclastogenic factors (E) in PDLCs. The PDLCs were pre-treated with Wnt5a siRNA (30 nM) or Wnt5 peptide (500 ng/mL) for 2 hours, post-incubated with Tβ4 peptide (1 μg/mL) and 200 μM H_2_O_2_ for 48 hours (A-E), and then conditioned medium (CM) was collected. The bar graph shows the fold increase in protein or mRNA expression compared with control. * Statistically significant differences compared with the control, *p*<0.05. # Statistically significant difference compared with the H_2_O_2_-treated group. The data presented were representative of three independent experiments.

**Fig 11:**
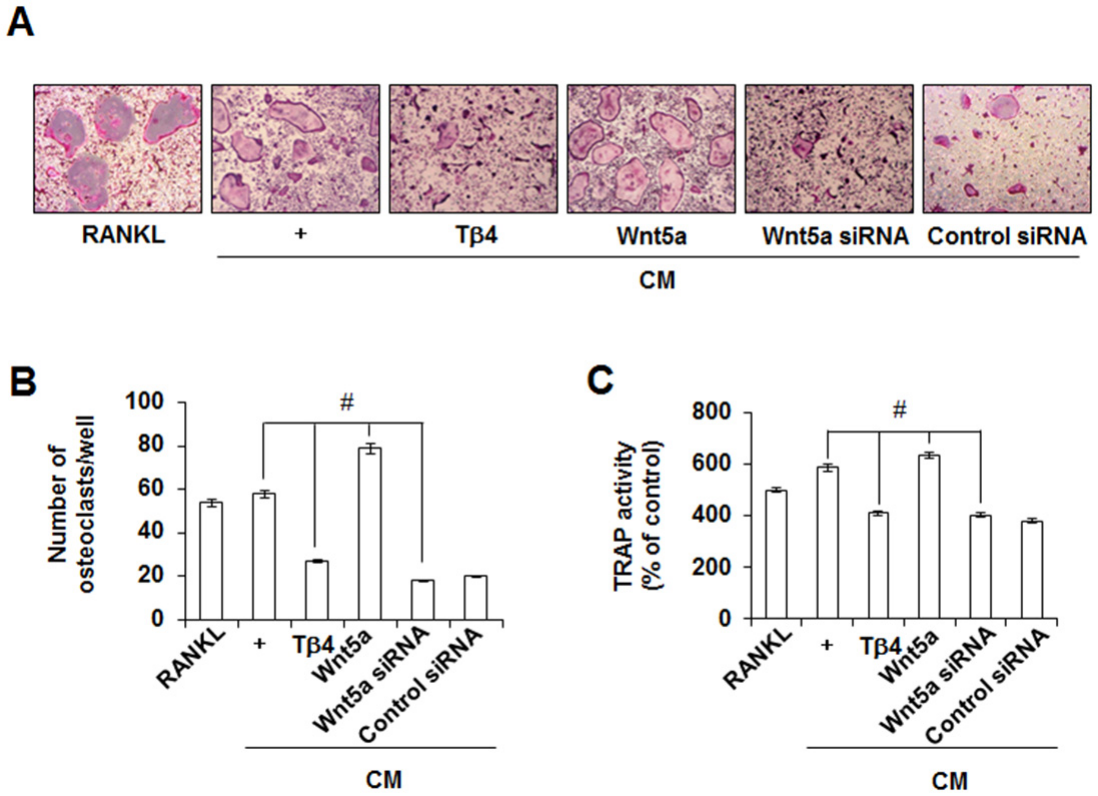
Effects Wnt5a siRNA and Wnt5a peptide on the Tβ4 peptide-mediated RANKL-induced osteoclastogenesis in mouse BMMs (A-C). The BMM cells were incubated with M-CSF (30 ng/mL) and RANKL (100 ng/mL) or CM collected from PDLCs. * Statistically significant differences compared with the control, *p*<0.05. # Statistically significant difference compared with the each group. The data presented were representative of three independent experiments.

To explore whether Tβ4 peptide-induced anti-inflammatory and anti-osteoclastogenesis were dependent on the up-regulation of Wnt5a, the effects of recombinant human (rh) Wnt5a (500 ng/mL) and Wnt5a-specific siRNA were assessed. Pretreatment of Wnt5a siRNA reversed the inhibitory effects of Tβ4 peptide on H_2_O_2_-induced iNOS and COX-2 expressions, NO and PGE_2_ productions, osteoclastogenic cytokines, and RANKL expression (Fig 10A–10E). In contrast, pretreatment with rhWnt5a enhanced the anti-inflammatory effects of Tβ4 peptide whereas control siRNA showed no effect on PDLCs. In accordance with anti-inflammatory results, Tβ4 peptide-suppressed osteoclast number and TRAP activity in BMM cells were reversed by exogenous treatment with Wnt5a siRNA but enhanced by rh-Wnt5a (Fig 11A–11C).

## Discussion

Oxidative stress is characterized by an accumulation of ROS and plays a key role in the progression of periodontal diseases [24]. Damage of tissues in inflammatory periodontal disease can be mediated by ROS resulting from the physiological activity of PMN during the phagocytosis of periodontopathic bacteria [27]. In addition, LPS from *Porphyromonas gingivalis* as well as hypoxia induces a NOX4-dependent increase in H_2_O_2_ release in PDLCs [28]. Furthermore, ROS such as H_2_O_2_ are small, diffusible, and ubiquitous molecules, can affect human PDLCs and gingival fibroblasts cell injury indirectly by enhancing pro-inflammatory factors such as cytokines, NO, PGE_2_, and ROS [29–31]. This ROS is known to stimulate osteoclast differentiation and participate in early signaling events associated with osteoclast activation for bone resorption [32]. Since LPS from *P*. *gingivalis* increases oxidative stress in PDLCs and contributes to periodontitis [28], human PDLCs treated with H_2_O_2_ may serve as an *in vitro* model relevant to periodontitis.

Tβ4 is the major monomeric actin-sequestering peptide in human tissues, and can bind globular actin (G-actin) in a 1:1 ratio and consequently involved in cytoskeletal regulation by inhibiting the polymerization of G-actin into fibrous actin (F-actin) [7]. In addition, Tβ4 is an ubiquitous naturally occurring molecule and is found at concentrations of 1 × 10^−5^ to 5.6 × 10^−1^ M in a variety of tissues and cell types, yet, no receptors for the protein have been identified [33]. A recent study suggests that internalization of exogenous Tβ4 is essential for its subsequent cellular functions [34]. Moreover, Tβ4 has been shown to be associated with, wound healing, hair growth, immunomodulation, and angiogenesis [7–9].

Tβ4 is not a thymus-specific peptide but also present in most tissue and all cells except red blood cells [35]. High amounts of Tβ4 were detected in human white blood cells, especially in neutrophils and in macrophages [34], expressed in developing mandible (embryonic day 12) [36] and hair follicles (HF) of mice [37]. In addition, the peptide is also detected outside cells, in blood plasma and in wound and blister fluids [34]. Although the mechanism(s) of action of exogenous Tβ4 on anti-inflammatory effects remains unclear, the high levels of Tβ4 present in human wound fluid (13 μg/mL) suggest its importance in wound healing or anti-inflammation [38]. However, the level of Tβ4 is variable (unchanged, decreased, and increased) in GCF or biopsied gingival tissue of periodontal patients [20, 21]. Based on the observations that Tβ4 has anti-inflammatory effects [11–14], the hypothesis is that Tβ4 regulates inflammatory mediators and osteoclastogenesis in osteolytic bone disease, such as periodontitis.

In this study, Tβ4 mRNA down-regulation was detected in *in vitro* in PDLCs stimulated with the ROS. This down-regulation of Tβ4 was also observed in GCF of periodontitis patient [19] and endotoxin-induced septic shock of rats [39]. ROS were generated predominantly by polymorphonuclear leukocytes (PMN) during an inflammatory response and involved in tissue destruction associated with periodontal diseases [40]. Thus, we chose to use ROS-stimulated PDLCs in this study since ROS, such as superoxide and H_2_O_2_, have been proposed as key players in bone resorption [41] and implicated in the pathogenesis of rheumatoid arthritis and periodontitis [29].

Exogenous Tβ4 can function like a hormone on cells in terms of its ability to modulate their biological behavior. Since one of the primary roles of Tβ4 in cells is the sequestration of actin monomers, and the protein is not secreted, previously indicated that it was unlikely that Tβ4 could have a hormonal function [42]. However, other studies have shown that the intracellular level of Tβ4 or its mRNA can be significantly and rapidly altered by external stimuli and that change in the level of Tβ4 often are correlated with cell differentiation [18, 43]. In the present study, exogenous Tβ4 peptide activate intracellular Tβ4, which results suggested that exogenous Tβ4 spontaneously enter the cytoplasm through rapid internalization, and acts their functions same as endogenous one [8, 18].

The full-length Tβ4 polypeptide has been shown to be effective in reducing inflammation [44]. It is also reported that only the 4-AA, amino-terminal peptide of Tβ4, known as Ac-SDKP, can block inflammation [45]. In this study, we used a synthetically human peptide produced copy of a naturally occurring, highly conserved 43-amino acid (MW = 4964 Da) water soluble acidic peptide, originally isolated from bovine thymus tissue [46]. This peptide is produced by Fmoc solid-phase peptide synthesis in accordance with the current Good Manufacturing Practice (cGMP) regulations (21 CFR 210 and 211) of the FDA [47]. An effective healer, Tβ4 can be administered topically on the surface of cells and systemically, through injection [9–11]. In this study, Tβ4 activation by Tβ4 peptide inhibited H_2_O_2_-induced production of NO and PGE_2_, expression of COX-2 and iNOS, and mRNA expression of TNF- α, IL-1β, -6, -8, and -17 in cultured PDLCs. These findings suggested that Tβ4 activation possessed anti-inflammatory activity in PDLCs. These results were consistent with previous *in vivo* and *in vitro* studies [9–15]. MAPK is a proline-directed serine/threonine kinase consisting of three-enzyme modules; its targets, inducing ERK, JNK and p38 kinases, are important in cellular signal transduction pathways and exert an anti-inflammatory response [48, 49]. NF-κB is a major transcription factor involved in the release of proteins that mediate the inflammatory response, and the degradation and phosphorylation of Iκ-Bα are necessary to release NF-κB from the cytoplasmic NF-κB/Iκ-Bα complex and allow its subsequent translocation to the nucleus of the cell [50]. In this study, Tβ4 peptide down-regulated the H_2_O_2_-triggered activation of the ERK and JNK MAPKs and the NF-κB in PDLCs. These results suggested that the ERK and JNK MAPKs and the NF-κB pathway may be involved in the anti-inflammatory effects of Tβ4 activation in PDLCs. Consistent with our findings, Tβ4 treatment decreased TNF-α-induced NF-κB activation in human corneal epithelial cells [51].

The RANKL and OPG have been identified as a key regulatory component of alveolar bone loss associated with inflammatory periodontal disease [52]. Moreover, PDLCs were shown to express several osteoclastogenic cytokines, including both OPG and RANKL [30, 31]. Our data demonstrated that Tβ4 peptide abolished H_2_O_2_-induced RANKL expression and restored OPG expression. Osteoclasts, bone-resorptive multinucleated cells derived from hematopoietic stem cells, are associated with osteolytic diseases. Furthermore, NFATc1, a master modulator of osteoclastogenesis, regulates target genes, such as cathepsin K and calcitonin receptor or Calcr [53]. In our *in vitro* study using BMMs, Tβ4 peptide directly and indirectly inhibited RANKL-induced osteoclast differentiation and expression of osteoclast markers, such as cathepsin-K, calcitonin receptor or Calcr, NFATc1, and RANK in BMM cells. These results indicated that Tβ4 was a key therapeutic target in controlling inflammation-induced bone loss.

MAPKs and NF-κB played pivotal roles in the development of osteoclasts downstream of RANK signaling [54]. In this study, we demonstrated that Tβ4 activation by Tβ4 peptide inhibited RANKL-induced p38, ERK, JNK MAPK, and NF-κB signaling pathways. These results suggested that Tβ4 activation might inhibit osteoclast differentiation via inhibition of the signaling cascades MAPK/NF-κB/NFATc1.

Recent reports have stated that inhibitors of Wnt signaling have emerged as promising strategies for bone disease and inflammatory diseases [26, 55]. Wnt5a, one of the most common Wnt molecules that activate the non-canoical pathway, binds to Fzd and its co-receptor, Ror2 [56]. In synoviocytes from rheumatoid arthritis patients, the expressions of Wnt5a and Frizzled5 (Fzd5) were significantly enhanced [25] and their blockades inhibited synoviocyte activation [55]. Recently, Wnt5a was highly expressed in synovial tissues in a mouse model of rheumatoid arthritis where inhibition of Wnt5a-Ror2 signaling suppressed bone loss [57]. Our data demonstrated that ROS up-regulated Wnt5a and its cell surface receptors, Frizzled and Ror2, as well as mRNA and protein expressions in time- and dose-dependent manners in PDLCs.

These results were in agreement with previous studies that showed Wnt5a expression can be induced in activated macrophages, endothelial cells, and bone marrow mesenchymal stem cells (BMSCs) after inflammatory stimulation [58, 59]. In addition, we found that the effects of Tβ4 peptide on H_2_O_2_-mediated induction of pro-inflammatory cytokines (NO, PGE_2_, TNF-α, IL-1β, IL-6, IL-8, and IL-17), the expression of inflammatory mediators (iNOS and COX-2), osteoclastogenic cytokines (cathepsin-K, calcitonin receptor or Calcr, NFATc1, and RANK), and osteoclastic differentiation, were reversed by exogenous treatment with Wnt5a siRNA but enhanced by rh-Wnt5a, suggesting that the anti-inflammatory and anti-osteoclastogenetic effects of Tβ4 activation were involved the Wnt5a-dependent signaling pathway. Similar to our results, Wnt5a knock-down markedly reduced cytokine/chemokine production induced by TNF in HDPCs [60].

Recently, therapeutic biomolecules such as growth factors provide great potential as an alternative therapeutic approach to traditional periodontal wound healing [61]. However, because of the short half-lives of growth factors and polynucleotides in the body and the necessity to deliver to specific target sites, those medicinal substances do not always exhibit the anticipated therapeutic potency and outcomes [62]. Thus, optimized delivery regimes and well-defined release kinetics appear to be logical prerequisites for safe and efficacious clinical application of biomolecules. For considering the application of Tβ4 in clinical trials, target cells of exogenous Tβ4 should be restricted to cells in the periodontal tissue.

In conclusion, this study is the first study to demonstrate that down-regulation of Tβ4 was observed in an *in vitro* model of H_2_O_2_-stimulated PDLCs. Tβ4 activation had anti-inflammatory effects via MAPK and NF-κB pathways in PDLCs and anti-osteoclastogenic effects via MAPK, NF-κB, and Wnt5a pathways in BMMs. These findings supported the fact that Tβ4 peptide could possibly be used in the development of a therapeutic drug for periodontitis and osteolytic disease.
